# S27. RELIABILITY OF SCHIZOPHRENIA DIAGNOSES IN CHILDREN AND ADOLESCENTS IN DENMARK

**DOI:** 10.1093/schbul/sby018.814

**Published:** 2018-04-01

**Authors:** Ditte Lammers Vernal, Anne Dorte Stenstrøm, Nina Staal, Anne Marie Raabjerg Christensen, Christine Ebbesen, Anne Katrine Pagsberg, Christoph Correll, René Ernst Nielsen, Marlene Briciet Lauritsen

**Affiliations:** 1 Aalborg University Hospital; 2 University Clinic Odense, University of Southern Denmark; 3 Child and Adolescent Mental Health Center, Mental Health Services, Capital Region of Denmark; 4 Center for Child and Adolescent Psychiatry, Aarhus University Hospital, Central Denmark Region; 5 The Zucker Hillside Hospital, Northwell Health; 6 Aalborg University Hospital, North Denmark Region

## Abstract

**Background:**

Schizophrenia in children and adolescents are diagnosed using the same criteria as for adults, but the assessment may be more complex due to both developmental issues, premorbid difficulties and a less elaborated symptomatic presentation. There is a great scarcity of studies looking into validity of schizophrenia in children and adolescents.

**Methods:**

We aimed to assess 1) the concordance and validity of schizophrenia register diagnoses among children and adolescents (early onset schizophrenia=EOS) in the Danish Psychiatric Central Research Register (DPCRR), and 2) the validity of clinical record schizophrenia diagnoses. Furthermore, to extract data from psychiatric records with confirmed schizophrenia in order to describe premorbid characteristics, history and symptomatology.

Psychiatric records from 200 patients with a first-time diagnosis of schizophrenia (F20.x) <18 years between 1994 and 2009 in the DPCRR was randomly selected for the study. The psychiatric records were evaluated by experienced clinicians according to ICD-10 criteria, using a predefined checklist. All records were assessed by two raters and inter-rater reliability was assessed.

**Results:**

We were able to retrieve 178 of the 200 psychiatric records. The mean age of patients was 15.2 years, and 56.2% were male. The register-based and clinical diagnosis matched in 158 cases. In the 10.2% registration errors, the records reported schizophrenia as a rule-out tentative diagnosis in the majority of cases. Among the 158 psychiatric records with a clinical diagnosis of schizophrenia, the raters confirmed 132 records (83.5%) as schizophrenia and a total of 145 records (91.8%) as in the schizophrenia-spectrum. Interrater reliability was substantial with Cohen’s kappa >0.78–0.83. Compared to diagnoses made in outpatient settings, EOS diagnoses during hospitalizations had fewer registration errors and a higher validity between raters’ diagnosis and clinical diagnosis.

Among the cases with EOS confirmed by raters, 85.8% had family history of mental disorders, 93.1% had experienced adverse life events during childhood with 46.9% having experienced trauma. Hallucinations were present in 76.9%, negative symptoms in 57.4% and formal thought disorder symptoms in 34%. Catatonic symptoms were described in 4.7% cases.

**Discussion:**

To our knowledge, the study is the largest to date investigating validity of schizophrenia diagnosed in children and adolescents in clinical settings. The study confirms assessment of schizophrenia in children and adolescents to be complex, especially in outpatient settings. All evaluations by raters were conducted by use of psychiatric records retrospectively. As the diagnoses were made 8 - 24 years ago, it is believed to be the best method, however, the possibility exists that some cases were not confirmed due to lack of adequate description of psychopathology in the records. Furthermore, the raters were not blinded to the diagnoses, as only patients with a register diagnosis of schizophrenia were included.

